# Preparation and Characterization of the MMT@Fe_3_O_4_@Ag Nanocomposite for Catalytic Degradation of Methyl Yellow: Reaction Parameters and Mechanism Based on the Artificial Neuron Network

**DOI:** 10.1021/acsomega.4c02497

**Published:** 2024-12-25

**Authors:** Türkan Altun, Musa Kazım Acar, Ilkay Hilal Gubbuk

**Affiliations:** † Department of Chemical Engineering, Konya Technical University, Konya 42150, Turkey; ‡ Department of Chemistry, Selcuk University, Konya 42130, Turkey

## Abstract

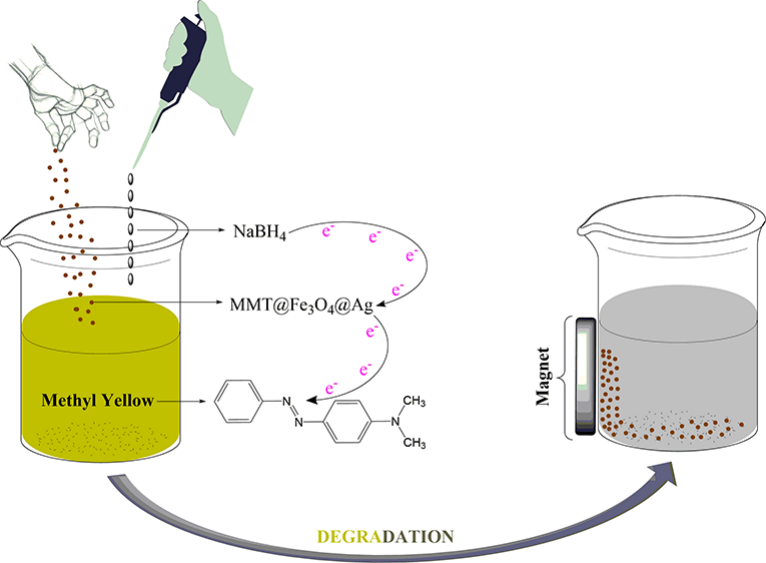

The montmorillonite@iron oxide@silver (MMT@Fe_3_O_4_@Ag) nanocomposite, which is recyclable and exhibits high catalytic activity, was evaluated for the degradation of methyl yellow (MY), a carcinogenic azo dye. For this purpose, MMT@Fe_3_O_4_ was first synthesized via the coprecipitation method and then Ag was doped to MMT@Fe_3_O_4_ via the chemical reduction method. MMT, MMT@Fe_3_O_4_, and MMT@Fe_3_O_4_@Ag were characterized by various techniques including scanning electron microscopy, Fourier transform infrared spectroscopy, X-ray diffraction, vibrating sample magnetometer, and thermal gravimetric analysis. The results illustrated that MMT@Fe_3_O_4_@Ag exhibited a higher catalytic ability than MMT@Fe_3_O_4_ toward decolorization of MY with a degradation efficiency of 100% in 10 min at pH 7.1 in the presence of sodium borohydride (NaBH_4_). Further, some parameters like the amount of NaBH_4_, initial dye concentration, and pH were also studied to determine optimum reaction conditions. MMT@Fe_3_O_4_@Ag could be easily separated and recycled from the reaction medium using an external magnet. Thus, the Ag-doped MMT@Fe_3_O_4_ nanocomposite proved to have good catalytic activity, high MY degradation rate and reusability, and easy separation and simple synthesis method. These properties make it a promising catalyst for the treatment of wastewater containing organic pollutants. In addition, artificial neural network (ANN) simulation, which is a mathematical model with an artificial intelligence algorithm, was used for the degradation process. This model was evaluated with the parameters used in the experiment as the input and output layers. Last, the degradation of MY with the synthesized catalyst into different products was demonstrated by high-performance liquid chromatography (HPLC) analysis.

## Introduction

Nanotechnology has an important place in many technological applications such as environmental remediation, cancer treatment, and food packaging in terms of environmentally healthier, more reliable, and reduced use of industrial chemicals. Therefore, nanotechnology is the controlled application of the properties of matter at the molecular level owing to nanoparticles or nanocomposites (mixture of two or more materials to give new properties to materials produced in the nanorange). In this context, the use of nanocomposites in the desired application areas for nanotechnology is of great importance.^1,2^ Due to their developable properties, nanocomposites are of great interest to researchers because they have wide usages in various fields like medicine,^3^ engineering,^4^ and food.^5^

Nanocomposites can be defined as composite materials in which nanosized reinforcements as fillers are dispersed within the base material as a matrix, which makes them superior to conventional materials. While three types of fillers as spheroid-like particulates, cylinder-like nanofibers (nanotubes), and flake-like (disk-like) platelets (nanolayers and nanoclays) are used in nanocomposites, the most common materials used as matrices are ceramics (e.g., alumina, glass, and porcelain), metals (e.g., titanium, iron, and magnesium), and polymers (e.g., epoxy, polyepoxide, nylon, and poly(ether imide)).^6^ As fillers, nanoclays have an important place due to their low environmental impact, partially low cost, and wide availability. Depending on their chemical composition and particle morphology, clays can be divided into classes such as kaolinite, smectite, halloysite, and chlorite. Montmorillonite (MMT) nanoclay (smectite), one of the most widely used clays among these types and always worthy of consideration, consists of alumina silicate layers.^7,8^ MMT is recognized by its structure occurring as a 2:1 sheet with one Al^3+^ octahedral sheet sandwiched between two Si^4+^ tetrahedral sheets. Its formula is (Na, Ca)_0.33_(Al, Mg)_2_(Si_4_O_10_)(OH_2_)·*x*H_2_O. The reason why MMT is preferred over other clay equivalents (kaolinite and illite) is that it has high cation exchange capacity and high net negative charge because of isomorphic substitution in the interlayer sheets and on broken edge regions of silica-alumina units.^9,10^ So, thanks to its distinctive features, it is widely used and evaluated as a work item in many fields such as in drug delivery,^11^ as a catalyst in H_2_ generation,^12^ and as an adsorbent for dye removal.^13^

MMT acts as a solid promoter in many chemical reactions, in both its natural and modified forms. There is also an improvement in the porosity and surface area of MMT during its modification with organic substances, acids, or transition metal nanoparticles. Since clays are suitable solid supports, they can be synthesized with transition metal nanoparticles and used as composite materials. Fe_3_O_4_ is more preferred as a nanomagnetic material to give magnetic properties to MMT.^14^

Today, magnetic nanoparticles (MNPs) are used in new potential applications such as biotechnology, ecology, and catalysis due to their improved thermophysical properties, functionality, high surface area, and good separability ability. Thus, MNPs as a suitable nanoadsorbent or nanocatalyst have been evaluated in dye degradation, removal of trace organic pollutants, and separation of toxic metals for years. Iron oxide is found in different forms in nature including maghemite (γ-Fe_2_O_3_), hematite (α-Fe_2_O_3_), and magnetite (Fe_3_O_4_). Among them, Fe_3_O_4_ is the most common iron oxide because of its low price and low toxicity. There are many methods for the synthesis of MNPs such as coprecipitation or reduction, hydrothermal synthesis, and thermal decomposition. However, as the bare Fe_3_O_4_ will create aggregation in the sample solution after synthesis, it must be modified and functionalized before desired application. For this purpose, MNPs are functionalized with organic materials such as polymers or inorganic materials such as gold, Ag, or oxide materials to provide a good dispersion in solution.^15−19^

It has been discovered that properties such as optical, catalytic, and biological properties of Ag nanoparticles (AgNPs) are related to their size, shape, and surface capping agents. However, aggregation of AgNPs is due to their size, and high surface energy will also restrict its catalytic activity. In addition, AgNPs will be difficult to separate from the reaction medium and will cause secondary pollution that prevents the use of AgNPs as catalysts. To avoid this situation, they can be functionalized with polymers,^20^ magnetic materials,^21^ and carbonaceous materials,^22^ which not only prevent aggregation but also have the desired catalytic activity.^23,24^

According to a study, more than 700 pollutants including petrochemical, personal care, textiles, and pesticides have been reported in the European region’s water ecosystem. The textile industry consumes 60% of paint production. Therefore, treatment of contaminated water with dye waste from the textile industry is a serious issue for our ecosystem.^25,26^ There are different synthetic dyes such as azo dyes, reactive dyes, acid dyes, and sulfur dyes used in the textile industry. Also, it is known that about 80% of these dyes are azo dyes. It has been documented that azo dyes containing one or more azo (−N=N−) and some functional groups in their structure have toxic, harmful, carcinogenic, mutagenic, and recalcitrant effects.^27−30^ For this reason, the degradation of azo dyes from wastewater becomes important in terms of environmental research.^31^ Dye removal methods can be analyzed in three categories as biological, chemical, and physical processes. While biological processes are evaluated as adsorption by algae degradation, enzyme degradation, and fungal cultures, chemical processes are considered as electrochemical degradation, oxidation, photochemical, and ultraviolet irradiation and physical processes are also evaluated as coagulation or flocculation, adsorption, nanofiltration, and ultrafiltration.^32,33^

MY, which is an azo amine dye with the chemical formula C_14_H_15_N_3_, is also known by the names of dimethylaminoazobenzene (DAB), *N,N*-dimethyl-*p*-phenylazoaniline, *para*-dimethylamino-azobenzene, *N,N*-dimethyl-4-aminoazobezene, 4-dimethylaminoazobenzene, *N,N*-dimethyl-4-(phenylazo)-benzenamine, benzeneazodimethylaniline, or butter yellow (Table S1). MY occurs as yellow leaf-shaped crystals at room temperature. While MY is soluble in chloroform, mineral acids, alcohol, ether, benzene, pyridine, oils, and petroleum ether, it is insoluble in water.^34^ MY, which has been banned since 1988 because of its carcinogenicity, was previously used as a food additive.^35^ Because it causes liver tumors in rats, bladder tumors in dogs, and cancer in mice, the carcinogenic properties of MY make it a dye to be emphasized.^36^ There are many studies in the literature on carcinogenicity,^34,35,37−39^ electrochemical degradation,^40,41^ catalytic degradation,^42^ photocatalytic degradation,^43^ and oxidation^44,45^ of MY. However, to date, no studies have been conducted on the catalytic degradation of MY in the presence of NaBH_4_.

ANN is an attempt to create artificial systems based on the computational model used by the nervous system that can process the information found in the human brain. In short, ANNs can be mentioned as a model that can imitate people’s thoughts and brain functions and draw a conclusion from the data they obtain. In the old-fashioned artificial intelligence approach, the task of programming the system algorithm is the responsibility of the programmer, whereas in the ANN approach, on the contrary, the system “learns” from the data.^46,47^ ANNs have the feature of learning the results obtained from the experiments and associating them with each other. Trained neural networks serve as a tool for highly accurate predictions of these results.^48^ The fact that ANNs are a science that focuses on adaptive data processing in situations where programming is difficult or very difficult makes it easier for researchers.^49^ Dye degradation modeling using ANN yielded high *R*^2^ values.^47^ There are many studies related to azo dye degradation in the literature using the ANN model. Some of these azo dyes are Reactive Red 141,^50^ Crystal Violet,^51^ Amido Black^52^ and Acid Black 1, and Acid Yellow 19.^53^

This paper has demonstrated for the first time and in a very short time the degradation of MY, whose chemical degradation in the presence of NaBH_4_ has never been studied in the literature. The aim of this study is to investigate the catalytic degradation of MY in the presence of NaBH_4_ with the MMT@Fe_3_O_4_@Ag nanocomposite. In this direction, the MMT@Fe_3_O_4_ magnetic-clay nanocomposite was first synthesized via the coprecipitation method. Further, Ag was doped via the chemical reduction method to this magnetic-clay nanocomposite. Further, the structure and morphology of synthesized MMT@Fe_3_O_4_ and MMT@Fe_3_O_4_@Ag were characterized by FTIR, XRD, SEM, EDS, VSM, Brunauer–Emmett–Teller (BET), and TGA. The degradation time of MY was observed at UV–vis after silver was doped to MMT@Fe_3_O_4_. Also, various parameters such as pH, amount of NaBH_4_, catalyst dosage, and initial dye concentration were studied for the degradation of MY in the presence of MMT@Fe_3_O_4_@Ag.

In addition, the degradation using the ANN method was compared both theoretically and experimentally. The use of the ANN method in this study, which has never been tried before in the degradation of MY in the presence of NaBH_4_, clearly demonstrated the novelty and originality of the study.

## Experimental Section and Methodology

### Preparation of MMT@Fe_3_O_4_

**Figure 1:**
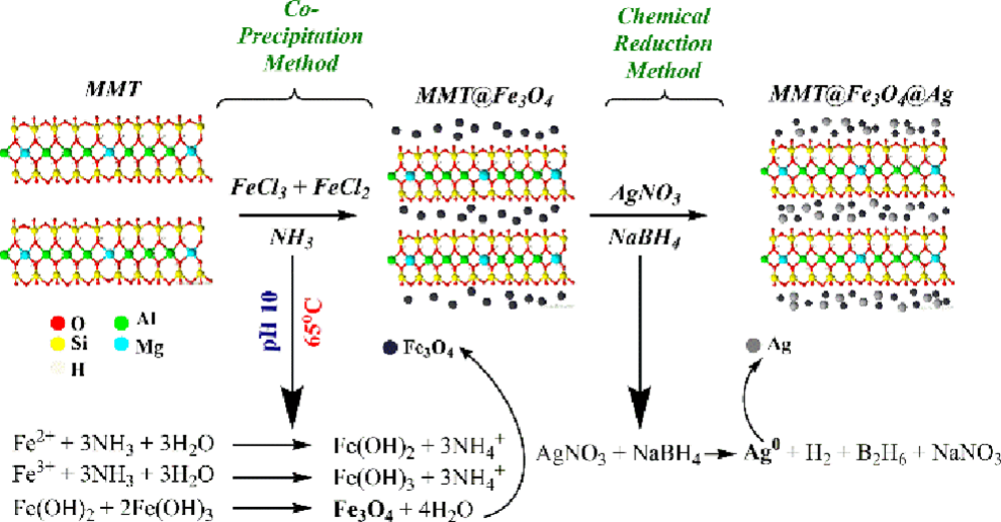
Detailed synthesis steps of MMT@Fe_3_O_4_ and MMT@Fe_3_O_4_@Ag.

The MMT@Fe_3_O_4_ magnetic-clay nanocomposite was prepared via the chemical coprecipitation method. The chemical reaction of the MMT@Fe_3_O_4_ synthesis is presented in Figure 1. A 1.67 g portion of FeCl_3_·6H_2_O and 0.43 g of FeCl_2_·4H_2_O were dissolved in 100 mL of deionized water. MMT (2.0 g) was added into 200 mL of deionized water, and the suspension was ultrasonicated for 30 min. The pre-prepared iron-containing solution (100 mL) was added to the MMT suspension (200 mL). After complete solubilization of the reactants, the final solution (300 mL) was heated to 65 °C during stirring at 400 rpm with the aid of a mechanical stirrer. After reaching this temperature, precipitant ammonia (25%) was added drop by drop until it reached pH 10.2. The final solution was stirred for an additional 1 h, and a dark brown precipitate originated at the bottom of beaker filtered by filter paper. After the obtained precipitate was filtered by filter paper, it was washed with deionized water for a few times. Then, it was dried under vacuum condition for 24 h at 65 °C.^54^

### Preparation of MMT@Fe_3_O_4_@Ag

The MMT@Fe_3_O_4_@Ag nanocomposite was prepared via the chemical reduction method (Figure 1). For this purpose, 0.5 g of MMT@Fe_3_O_4_ was dispersed in 200 mL of deionized water, and it was kept in an ultrasonic bath for 30 min. Afterward, 30 mg of AgNO_3_ was dispersed in 20 mL of deionized water. Prepared AgNO_3_ solutions (20 mL) were added into the solution of MMT@Fe_3_O_4_ (200 mL). This mixture was vigorously stirred for 2 h at room temperature, and 0.1 g of NaBH_4_ was added into the final solution (220 mL) during stirring. After this process, the mixture was filtered and washed with deionized water. The obtained precipitate was dried under vacuum for 24 h at 65 °C.^54^

### Characterization

FTIR spectra of MMT, MMT@Fe_3_O_4_, and MMT@Fe_3_O_4_@Ag were determined using a Bruker Vertex 70 spectrometer and were measured from 4000 to 400 cm^–1^. The analyses of crystalline phases were performed by XRD in a GNR–APD 2000 Pro X-ray Diffractometer with CuKα (λ = 1.542 Å) radiation, a 2θ range of 2–80°, and a step size of 0.02°. The morphologies of the materials were investigated using SEM (Hitachi-SU 1510) equipped with energy disperse spectroscopy (EDS) for the analysis of the surface element composition. VSM (Cryogenic Limited PPMS) was used for the magnetization measurement of the MMT@Fe_3_O_4_@Ag nanocomposite at room temperature. BET analysis by N_2_ adsorption/desorption (QuadraSorb Station 1 Quantachrome, degassed at 300 °C) at liquid nitrogen temperature (77.4 K) was performed to obtain the specific surface area of the materials. The thermal resistance and stability of the nanoparticle were investigated using TGA (Setaram Labsys Evo). UV–visible spectral measurements for degradation experiments of MY were carried out using a Shimadzu UV-1700 UV–vis working in the range of 200–800 nm.

The point of zero charge (pH_pzc_) is essential in determining the MMT@Fe_3_O_4_@Ag nanocomposite’s surface properties. To determine the pH_pzc_ value of the MMT@Fe_3_O_4_@Ag nanocomposite, the batch equilibration technique described by Stoia et al. was used.^55^ For this purpose, 25 mL of 0.01 mol/L NaCl solution (in six different flasks) was adjusted to pH 2, 4, 6, 8, 10, and 12 using 0.1 mol/L NaOH and 0.1 mol/L HNO_3_ solutions. Fifty milligrams of MMT@Fe_3_O_4_@Ag was then added to each solution and stirred to equilibrate with a magnetic stirrer (150 rpm) for 24 h at 25 °C. MMT@Fe_3_O_4_@Ag was magnetically separated from the aqueous phase after stirring. The final pH of the solution (pH_fin_) was measured, and the difference (ΔpH) between initial (pH_in_) and final pH (pH_fin_) was plotted against the pH_in_ values. The value where these two points are zero is defined as pH_pzc_, and it was calculated as 5.62 for MMT@Fe_3_O_4_@Ag (Figure S1).

### Catalytic Degradation of MY

For catalytic degradation of MY, MMT@Fe_3_O_4_ and MMT@Fe_3_O_4_@Ag nanocomposites were evaluated separately in terms of investigation of the catalytic activity of Ag. Accordingly, a solution of MY (13.5 mg/L, 100 mL) was prepared in 1:1 (v/v) methanol/water. For reduction of MY, a solution of NaBH_4_ (0.01 mol/L, 25 mL) was prepared freshly for each experiment. A 3.5 mL UV cuvette was used to perform all catalytic experiments at room temperature. To a mixture containing 3 mL of MY and 0.375 mL of NaBH_4_ in the UV cuvette, 7.5 mg of MMT@Fe_3_O_4_ and MMT@Fe_3_O_4_@Ag was added separately. The change in intensity of the absorption peak at 444 nm was monitored at UV–vis. In addition to these, various parameters including pH (3.2–10.1), catalyst dosage (0.44–3.11 g/L), initial dye concentration (9–36 mg/L), and the amount of NaBH_4_ (0.125–0.425 mL) were investigated.

### ANN Modeling

ANN analysis calculations were performed using the mathematical software-based computer program MATLAB (R2022b). Back-propagation, a widely used learning algorithm, is an algorithm for ANN training that redistributes error through iteration. The main back-propagation algorithm used for this study was chosen as the Levenberg–Marquardt algorithm. The ANN model was composed of input (independent variables), hidden, and output layers (dependent variables). While the number of input and output neurons is determined according to the nature of the problem, the hidden layer consists of artificial neurons interconnected with associated weights (wi) and biases (bi).^56−58^ Solution pH, MMT@Fe_3_O_4_@Ag nanocomposite dosage, initial dye concentration, reaction time, and the amount of NaBH_4_ were taken as the input layer, and the percentage of MY dye degradation was taken as the output layer. Each data set was prepared in MS Excel worksheet and transferred to the MATLAB environment. The data set was divided into three clusters for training (70%), validation (15%), and testing (15%).

To avoid numerical overflows, the results obtained from the experimental data were normalized between 0 and 1. The normalization method was carried out using eq 1:
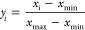
1where *y*_*i*_ is the normalized value of *x*_*i*_, and *x*_max_ and *x*_min_ are the maximum and minimum values of *x*_*i*_, respectively. The process of evaluating the results of the different networks was found with the formulas of mean square error (MSE) and coefficient of determination (*R*^2^) specified in eqs 2 and 3:
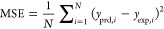
2
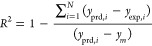
3where *N*, *y*_prd,*i*_, *y*_exp,*i*_, and *y*_m_ are the number of data, the *i*th model-predicted value, the *i*th experimental value, and the average of the experimental values, respectively.^59^

### HPLC Method

The species obtained from the degradation experiments under optimum conditions were analyzed by high-performance liquid chromatography (HPLC, Shimadzu Prominence-i series LC2030-3D Model). It was proved by HPLC analysis that MY reacted at the end of the degradation experiments and transformed into different products. A LiChrosorb RP-18, 7 pm column (25 cm × 4.0 mm I.D) was used in the HPLC system. The mobile phase was methanol–water (90:10, v/v), the injection volume was 10 μL, and the flow rate was 1 mL/min. The column temperature was set to 40 °C.^60^

### Stability and Reusing of the MMT@Fe_3_O_4_@Ag Nanocomposite

After the catalytic degradation reactions were completed, the MMT@Fe_3_O_4_@Ag nanocomposite was separated by an external magnet and washed several times with ethanol and deionized water. Then, the recovered dried nanocomposite under vacuum was used for the next cycle.

### Leach Process

The metal leaching amount for the synthesized MMT@Fe_3_O_4_ and MMT@Fe_3_O_4_@Ag catalysts in the degradation process was determined by a PerkinElmer/ICP OES OPTIMA 2100 DV.

## Results and Discussion

### Characterization of Nanocomposites

Since the nanocomposite used in this study is the same as the nanocomposite we used in our previous study, characterization graphs can be accessed from our previous study.^54^

The functional groups of MMT, MMT@Fe_3_O_4_, and MMT@Fe_3_O_4_@Ag were determined using FTIR. The FTIR spectrum of MMT showed stretching vibration peaks at 3622 and 3409 cm^–1^ due to the −OH group in the hydration water, the vibration absorption peak at 1628 cm^–1^ for H–O–H bending arising from water molecules in the interlayer of MMT, the absorption peaks, the characteristic peaks of MMT at 1032 cm^–1^ for Si–O–Si stretching and at 795 cm^–1^ for Si–O–Al stretching vibration, the absorption peaks at 919 cm^–1^ due to Al–O and at 831 cm^–1^ for (Al, Mg)–OH vibration modes, and the bond at 518 cm^–1^ indicated in the MMT@Fe_3_O_4_ spectrum for Si–O–M stretching vibration, where M could be Al, Mg, or Fe. In addition, there is an absorption peak at 459 cm^–1^, which proves the presence of the Fe–O bond in the MMT@Fe_3_O_4_ structure. After Ag is doped into the structure of MMT@Fe_3_O_4_, it presents the peak carbonyl (C=O) stretching observed at 1738 cm^–1^, while the peak at 1368 cm^–1^ can be associated with the C–H group.^54^ The FTIR spectra of MMT@Fe_3_O_4_ and MMT@Fe_3_O_4_@Ag nanocomposites obtained after MY degradation were compared with the FTIR spectra of MMT@Fe_3_O_4_ and MMT@Fe_3_O_4_@Ag nanocomposites obtained before the degradation reaction (Figure S2). No visible change was observed in the FTIR spectra of the nanocomposites.

The crystalline patterns of MMT, MMT@Fe_3_O_4_, and MMT@Fe_3_O_4_@Ag samples were analyzed by the X-ray diffraction pattern. MMT exhibited major dominant peaks at 2θ values of 17.7, 19.8, 29.8, 35, 55, and 62° (ICDD: 98-016-1171). The XRD diffraction of cubic Fe_3_O_4_ observed at around 2θ values of 30, 35.06, 39.5, 56.4, and 68° (ICDD: 98-019-1822) corresponds to (220), (311), (400), (511), and (440) Bragg reflections, respectively. The XRD patterns of MMT and MMT@Fe_3_O_4_ may not differ perceptibly due to the similarity of their characteristic peaks in both. However, it has been proven by other characterization methods that Fe_3_O_4_ is loaded onto the MMT surface. By adding AgNPs into MMT@Fe_3_O_4_, it was observed that new peaks were formed in the XRD pattern of the MMT@Fe_3_O_4_@Ag nanocomposite. These diffraction peaks observed for planes (111), (200), (220), and (311) of 2θ values of 38.1, 44.3, 64.7, and 77° validated the presence of AgNPs (ICDD: 98-018-0879). The characteristic Bragg peaks at (111), (200), (220), and (311) represent the face-centered cubic structure of Ag nanoparticles. This means that AgNPs crystallized well on the structure of MMT@Fe_3_O_4_. The average crystal size of the MMT@Fe_3_O_4_@Ag nanocomposite calculated using the Debye–Scherrer formula was 15 nm.^54^ Additionally, the XRD patterns of Fe_3_O_4_ is given in Figure S3, and XRD patterns of MMT@Fe_3_O_4_ MMT@Fe_3_O_4_@Ag nanocomposites obtained before and after MY degradation are given in Figure S4.

**Figure 2:**
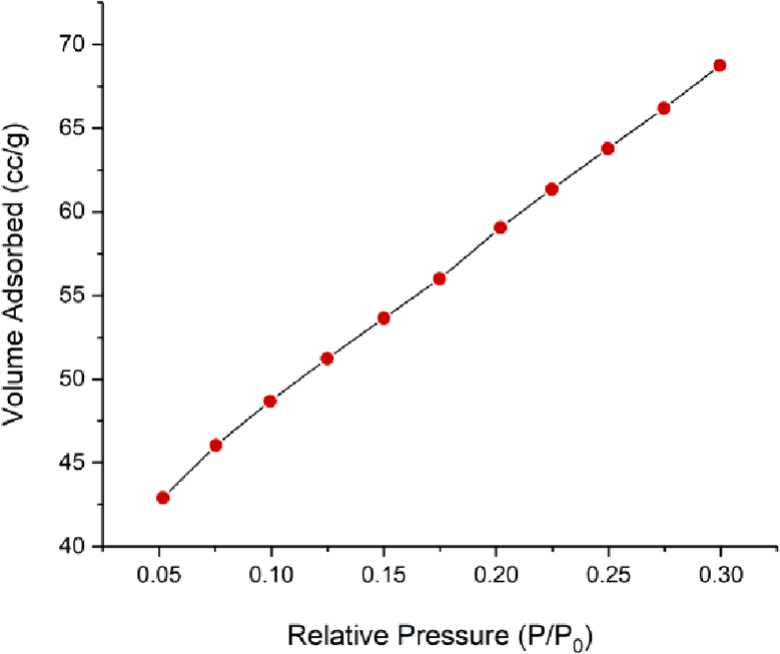
BET adsorption isotherm of MMT@Fe_3_O_4_@Ag.

MMT has a surface area of 210.037 m^2^/g and a pore volume of 0.374 cc/g. After the surface of MMT was loaded with Fe_3_O_4_, a decrease in pore volume and specific surface area of MMT was observed due to addition of Fe_3_O_4_ in pores or on the surface of MMT. Consequently, it showed that the specific surface area and pore volume of MMT reduced to 179.412 m^2^/g and 0.340 cc/g, respectively. Similarly, AgNPs gave rise to intense occupation of the surface of MMT@Fe_3_O_4_, which indicates a decrease in pore volume and specific surface area of MMT@Fe_3_O_4_.^54^ The BET adsorption isotherm of MMT@Fe_3_O_4_@Ag is given in Figure 2.

The magnetic measurement of the MMT@Fe_3_O_4_@Ag nanocomposite was investigated by VSM in the applied field ranging from −10000 to +10000 Oe at room temperature. The magnetic saturation (*M*_s_) value of MMT@Fe_3_O_4_@Ag was measured at 4.8 emu/g^–1^. This value demonstrates the magnetic nature of MMT@Fe_3_O_4_@Ag, indicating that it can be easily separated from the suspension using an external magnet after the reaction. The *M*_s_ value obtained from this study is lower than the experimentally determined 30–50 emu/g^–1^ value of MNPs synthesized by the coprecipitation method. This phenomenon is likely due to nonmagnetic AgNPs surrounding the MNPs.^54^

SEM images of MMT, MMT@Fe_3_O_4_, and MMT@Fe_3_O_4_@Ag to evaluate their morphology and, in addition, the EDX spectrum and elemental mapping were obtained to further confirm the composition and surface chemical state of MMT@Fe_3_O_4_@Ag. It was seen that after Fe_3_O_4_ and AgNPs were loaded into MMT, there was no significant change in its structure, which confirms that AgNPs and Fe_3_O_4_ were synthesized with a good distribution in the MMT structure. The existence of Ag, Fe, and Si elements in the structure of MMT@Fe_3_O_4_@Ag and distribution of these elements were clearly indicated by elemental mapping. When the EDX measurement was examined, the percentage of Si from MMT was 76.13 wt %, while the percentages of Fe and Ag were 9.36 and 0.96 wt %, respectively.^54^

**Figure 3:**
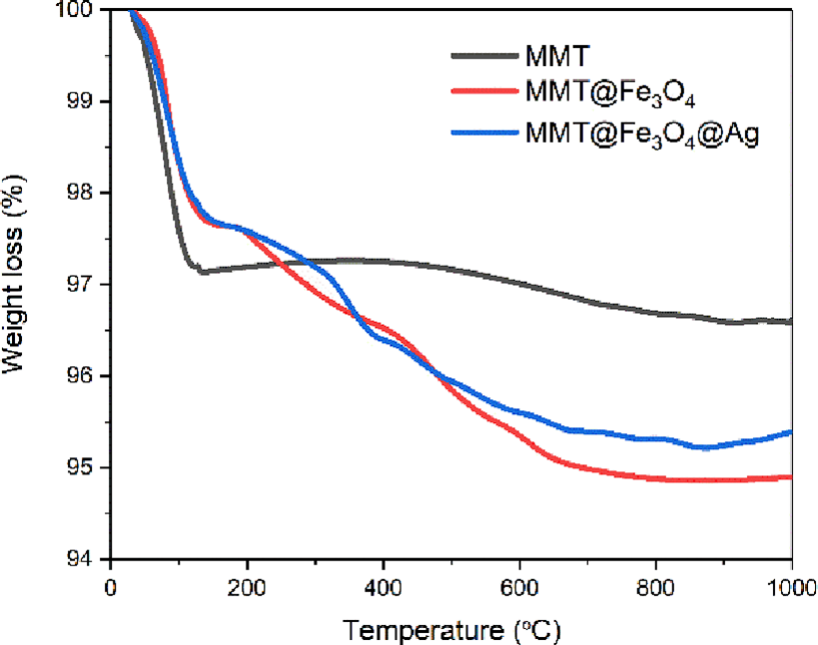
TG curves of MMT, MMT@Fe_3_O_4_, and MMT@Fe_3_O_4_@Ag.

The TG curves of MMT, MMT@Fe_3_O_4_, and MMT@Fe_3_O_4_@Ag are presented in Figure 3. During thermal analysis, the overall residues from the sample were 96.6, 94.9, and 95.4% for the MMT, MMT@Fe_3_O_4_, and MMT@Fe_3_O_4_@Ag samples, respectively. Since MMT was clay in these samples, it served as a greater barrier to the volatile products produced during the decomposition of the sample. As a result of the doping of MMT with Fe_3_O_4_ and Ag, a decrease in mass loss occurred for both nanoparticles depending on the temperature. This indicates an increase in material thermal stabilization.^61,62^

**Figure 4:**
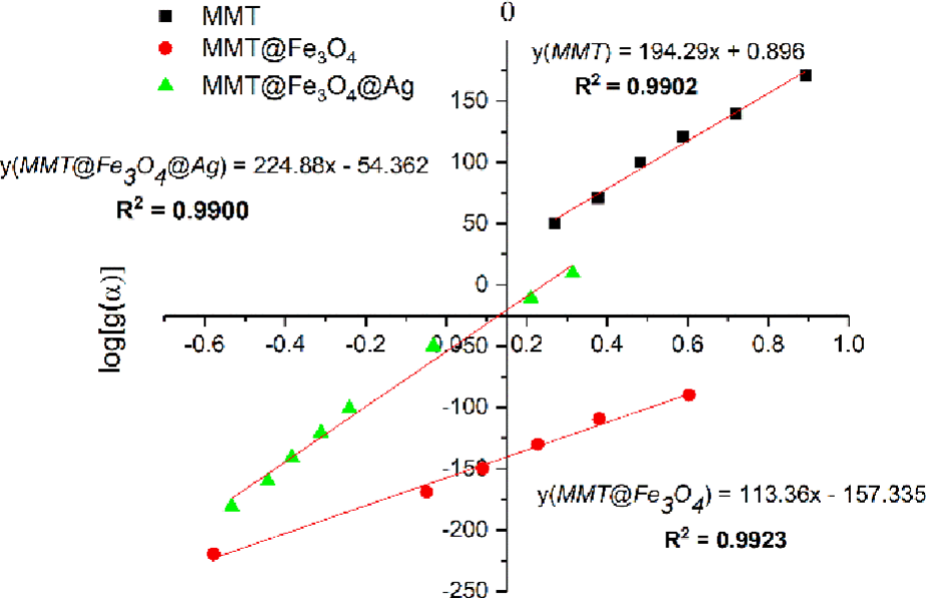
HM plots of MMT, MMT@Fe_3_O_4_, and MMT@Fe_3_O_4_@Ag materials.

In this study, the detailed investigation of the thermal decomposition of MMT, MMT@Fe_3_O_4_, and MMT@Fe_3_O_4_@Ag materials is mainly discussed. From the results of the thermogravimetric analysis, the kinetic parameters were determined using the Horowitz–Metzger (HM) equation. Based on the following equations, different thermodynamic parameters were calculated in this study. The thermal stabilities of MMT, MMT@Fe_3_O_4_, and MMT@Fe_3_O_4_@Ag were analyzed using thermogravimetric analysis for all three substances as shown in Figure 4.

In this study, activation energy (*E)*, preexponential factor (*A*), and thermodynamic parameters *(*Δ*G*,Δ*H*, andΔ*S*) were calculated using the HM equation. The degradation of the kinetic complex was investigated for the compounds MMT, MMT@Fe_3_O_4_, and MMT@Fe_3_O_4_@Ag. The degree of conversion α is defined as the ratio of the actual weight loss to the total weight loss. α is calculated according to eq 4:
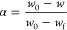
4where *w*_0_ is the initial mass of the sample (g), *w* is the mass of the sample at a time and temperature *T* (K), and *w*_f_ is the final mass of the sample. The rate of the degradation process can be defined by eq 5 as the product of two separate functions, temperature and fractional transformation.^63^
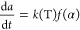
5where α is the fraction decomposed at time *t* (eq 7), *k*(*T*) (eq 6) is the temperature-dependent function, and *f*(α) (eq 6) is the transformation function dependent on the mechanism of degradation.
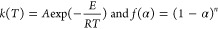
6then
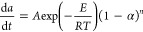
7

Using the HM method for *n* = 1 (*n*, reaction order)
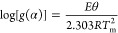
8Here, *R* is the gas constant and θ = *T – T*_m_, where *T*_m_ is the temperature of maximum reaction rate and *T* is the temperature in Kelvin at any instance (eq 8). A plot of log[*g*(α)] versus θ (Figure 4) should give a straight line whose slope is .^64^ The preexponential factor (*A*) is calculated by using eq 9:
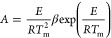
9Here, β is the heating rate (K min^–1^).

The values obtained for *E* and *A* of three functionalized materials have been calculated and are shown in Table 1.

**Table 1:** Kinetic and Thermodynamic Parameters of MMT, MMT@Fe_3_O_4_, and MMT@Fe_3_O_4_@Ag Molecules Determined Using the HM Equation (eq 8)

molecule	*E* × 10^8^ (J/mol)	*A* × 10^3^ (s^–1^)	Δ*S* (J/mol K)	Δ*H* × 10^8^ (kJ/mol)	Δ*G* (kJ/mol)
MMT	5.30	9.77	–288.8	5.14	5.30
MMT@Fe_3_O_4_	5.21	2.92	–301.2	4.53	5.21
MMT@Fe_3_O_4_@Ag	7.28	14.6	–286.2	4.58	7.28

The entropy of activation Δ*S* is calculated using eq 10:
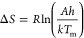
10where *k* is the Boltzmann constant and *h* is the Planck constant. The enthalpy activation Δ*H* and Gibbs free energy Δ*G* are calculated from eqs 11 and 12.
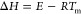
11
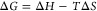
12

Due to the covalent character of the bonds of intermolecular bonds, the activation energy *E* value was high, indicating the high stability of all three metals. The value of Δ*G* was positive from each step and had the highest value for MMT@Fe_3_O_4_@Ag. The negative sign of the Δ*S* values, which causes the Δ*G* values to increase in the final step, obtaining MMT@Fe_3_O_4_@Ag, indicates the nonspontaneous nature of the steps. The endothermic nature of the steps is reflected by the positive sign of the Δ*H* values.^65^

### Catalytic Activity of MMT@Fe_3_O_4_ and MMT@Fe_3_O_4_@Ag for Degradation of MY

To investigate the catalytic activity of Ag, MMT@Fe_3_O_4_ and MMT@Fe_3_O_4_@Ag nanocomposites were first evaluated in the degradation of MY in the presence of NaBH_4_ at room temperature and pH 7.2. MY exhibits a characteristic absorption band at 446 nm.^66^ The absorption peak at 446 nm stems from a conjugated structure formed by the azo bond under the strong influence of the electron-donating dimethylamino group.^67^

**Figure 5:**
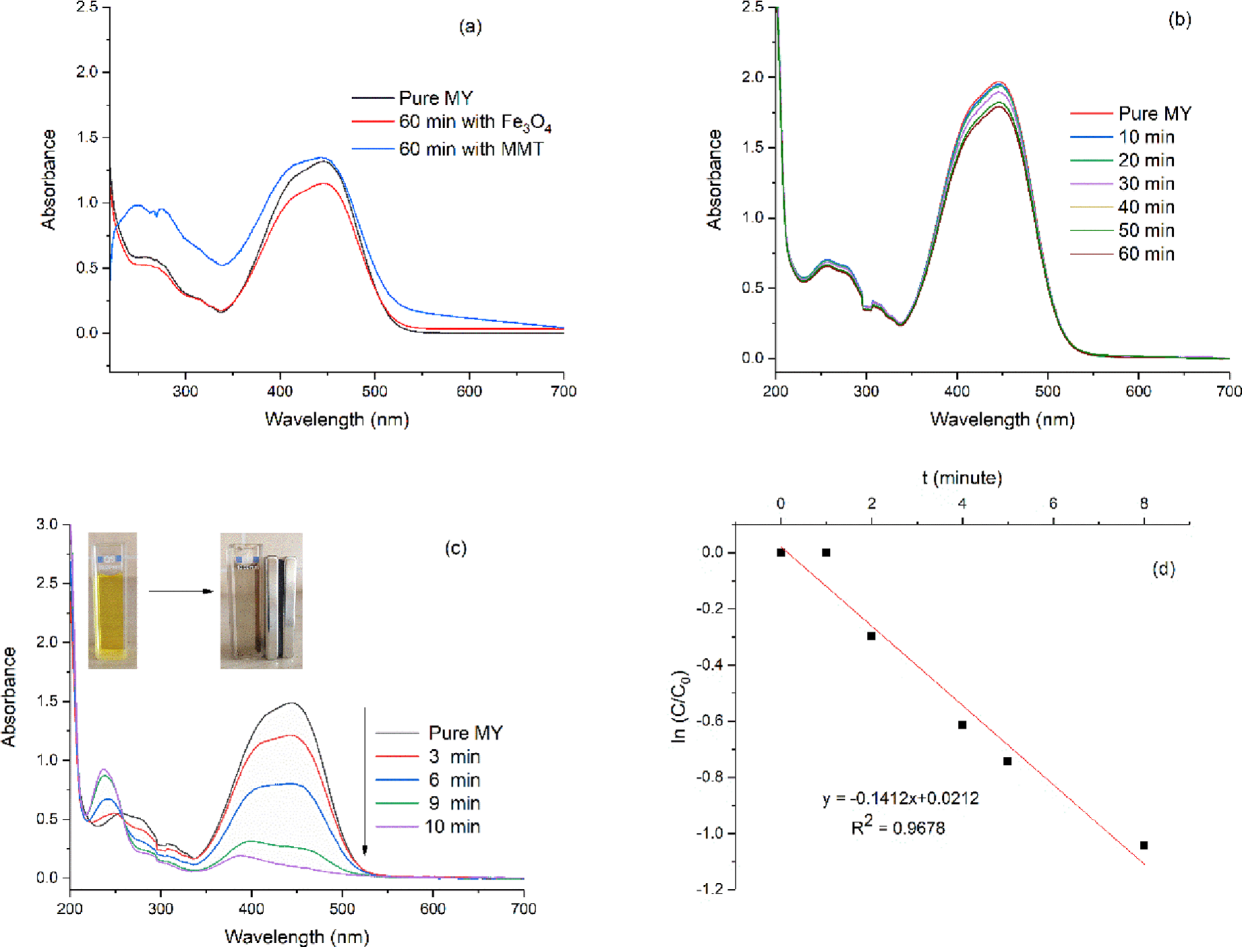
Degradation of MY in the presence of (a) MMT and Fe_3_O_4_ and (b) MMT@Fe_3_O_4_ and (c) MMT@Fe_3_O_4_@Ag [reaction conditions: MY = 13.5 mg/L, pH = 7.2, catalyst dosage = 2.22 g/L, and NaBH_4_ = 0.375 mL] and (d) plot of ln(*C*_*t*_/*C*_0_) versus time for degradation of MY.

The catalytic degradation reaction of MY was monitored in UV–vis at wavelengths in the range of 200–700 nm. To monitor the degradation of MY by UV–vis, the peak density at 446 nm, which indicates the yellow color and degradation of the MY solution, is taken into account. UV–vis spectra were monitored for the MY degradation of MMT and Fe_3_O_4_ separately for 60 min (Figure 5a). The results obtained had negligible degradation percentages, as can be seen from the graph. Figure 5b shows the UV–vis spectra of degradation of MY (13.5 mg/L) in the presence of MMT@Fe_3_O_4_ at a NaBH_4_ concentration of 0.1 mol/L, while Figure 5c shows UV–vis spectra of the degradation of MY in the presence of MMT@Fe_3_O_4_@Ag under the same conditions. It is clearly seen that the Ag-doped nanocomposite completely degraded MY within 10 min, the peak at 446 nm was zeroized, and yellow color of the dye vanished completely as the reaction time progressed, whereas MMT@Fe_3_O_4_ degraded only 10% of MY, although the reaction time advanced to 60 min. After complete degradation of MY, the MMT@Fe_3_O_4_@Ag nanocomposite was easily separated from the suspension by an external magnet.

### Kinetic Study and Mechanism of MY Degradation

In this study, the kinetic study of the catalytic reduction reaction of MY on a Ag-doped nanocomposite was investigated. The rate of degradation of organic dye molecules is usually expressed by the so-called pseudo-first-order kinetics derived from the Langmuir–Hinshelwood mechanism when the concentration of the reducing agent (NaBH_4_) is greater than the concentration of the dye during the degradation reaction.^68,69^

The rate constant for degradation of MY was calculated by the pseudo-first-order kinetic rate law (eq 13) given below:
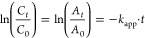
13Here, *k*_app_ is the pseudo-first-order rate constant for degradation of dyes, *C*_*t*_ represents concentration of MY at any time and *C*_0_ represents the initial concentration of MY, and *A*_*t*_ indicates the absorbance of MY (λ_max_ = 446 nm) at any time and *A*_0_ indicates the absorbance of MY (λ_max_ = 446 nm) at the beginning time.^71^ When Figure 5d is examined, the apparent reaction rate constant value obtained for degradation of MY on the Ag-doped nanocomposite was calculated as 0.1412 min^–1^ from the slope of the graph. Since MY has not been degraded with NaBH_4_ so far, comparison with other studies has been made with studies involving different degradation methods (Table 2). As can be seen from the table, degradation times of MY were found to be quite long compared to those in our study.

**Table 2:** Comparison of MY with Other Studies

dye	catalyst	degradation method	reaction time (minute)	reference
MY	P25 Degussa	photocatalytic degradation	over 150	(43)
	flexible multilayered triboelectric nanogenerator (FM-TENG)	electro-Fenton degradation	120	(41)
	g-C_3_N_4_-Co_3_O_4_-V_2_O_5_	photocatalytic degradation	80	(70)
	Pd/HAP/Fe_3_O_4_	catalytic degradation	80	(42)
	precharge-injected multilayer-linkage triboelectric nanogenerator (PMLL-TENG)	electrochemical oxidation degradation	800	(40)
	MMT@Fe_3_O_4_@Ag	catalytic degradation	10	our study

**Figure 6:**
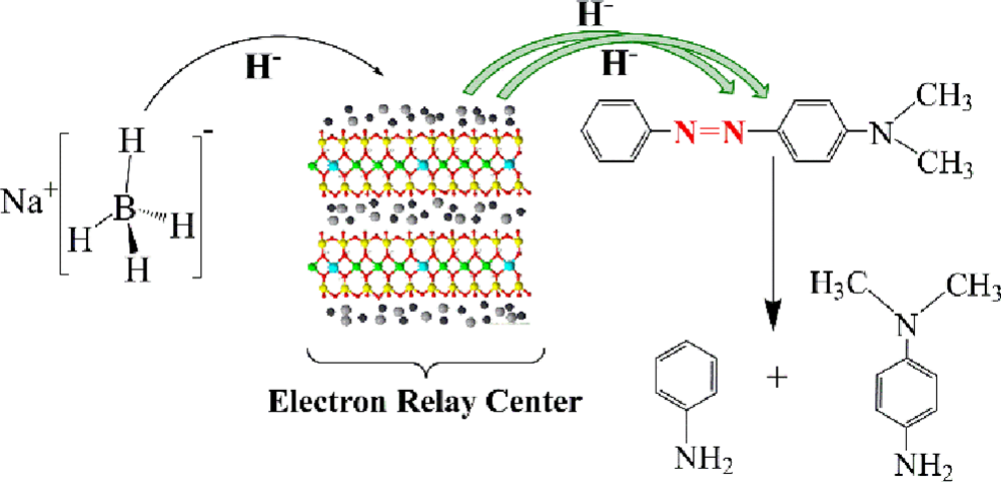
Breaking of the azo bond and degradation mechanism of MY with BH^–^ and MMT@Fe_3_O_4_@Ag.

Dyes containing at least one chromophore, such as azo, nitro, and anthraquinone, absorb light in the visible region (350–700 nm), which indicates that they have a wavelength of maximum absorption (λ_max_) and therefore are colored. These properties allow them to be monitored with UV–vis. Along with chromophores, they also have oxochromes known as color-enhancing groups such as −COOH, −SO_3_H, and −OH groups, which affect the solubility of many dyes. It is known that if any of the groups or bonds here are affected, the color of the dye is removed.^67,71^ Within this scope, the mechanism of degradation of MY through the MMT@Fe_3_O_4_@Ag nanocomposite in the presence of NaBH_4_ is illustrated in Figure 6. In this mechanism, while the MMT@Fe_3_O_4_@Ag nanocomposite plays a role as the electron relay center, NaBH_4_ and dye molecules act as the electron donor and acceptor, respectively. Hydrogens liberated by BH^–^ ions carry electrons on the surface of the nanocomposite to break the azo bond in MY molecules. Thus, the process called the electron transfer system progresses. The end of this process occurs when MY molecules gain electrons and form the reduced form of the dye. Electron transfer from BH_4_^–^ ions causes BO_3_^3–^ formation.^69^ The breakdown of the azo double bonds (−N=N−) of MY results in degradation products. These are aniline and *N*,*N*-dimethyl-*p*-phenylenediamine. Consisting of −NH_2_ and the benzene ring, aniline is observed at 230 nm in UV–vis, which is the characteristic absorption band of the benzene ring.^72,73^ In our work, we observed aniline at 237 nm, and Figure 5b presents the new band at 237 nm of aniline after degradation of MY. The intensity of this band increased as the reaction time progressed.

### Effect of the Solution pH on Degradation of MY

**Figure 7:**
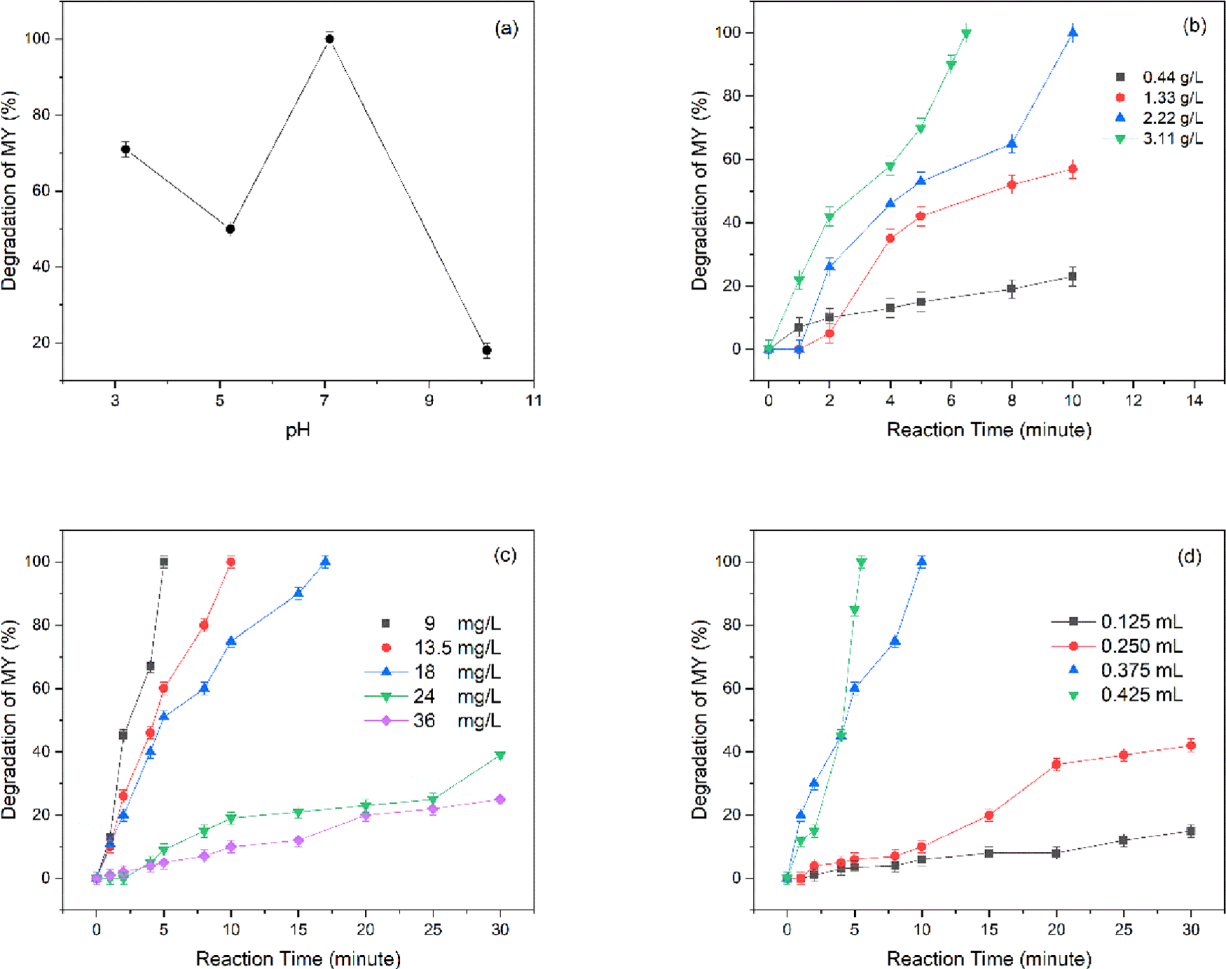
Effect of (a) pH, (b) MMT@Fe_3_O_4_@Ag dosage, (c) initial dye concentration, and (d) amount of NaBH_4_ on degradation of MY.

The pH value of the solution is of critical importance for the system under consideration as it will directly affect two different processes: (i) the surface charge of the nanocomposite in solution depending on the pH_pzc_ value and (ii) the degradation of the dye molecules on the nanocomposite because of the change in the surface charge. For this study, 4 different pH values as 3.2, 5.2, 7.1, and 10.1 were examined. The obtained results are given in Figure 7a. In addition, the change of the *k*_app_ constant value depending on the solution pH value is given in Figure S5. In a system containing 13.5 mg/L MY, 2.22 g/L MMT@Fe_3_O_4_@Ag, and 0.375 mL NaBH_4_ in 10 min, the degradation of MY reached 100% at pH 7.1. In addition, the highest *k*_app_ value was obtained at this pH under the same conditions. Figure S6 shows the acid–base and tautomeric equilibria of MY reported by Tawarah and Abu-Shamleh.^66^ In their study, it was reported that the alkali form obtained by adding NaOH to the MY solution started the aggregation process and that a decrease in the maximum absorption intensity of MY was observed depending on the time. On the other hand, with the decrease in pH (in the acidic media), the azonium-ammonium tautomer of structures B, C, and D in Figure S6, which are the monoprotonated forms of MY, appeared.^66,74^ The pH_pzc_ value of the MMT@Fe_3_O_4_@Ag nanocomposite was found as 5.62 (Figure S1). While the surface of the nanocomposite is positively charged below this pH value (pH < pH_pzc_), it becomes negatively charged above it (pH > pH_pzc_).^55^ Accordingly, in acidic media (pH 3.2 and 5.2), the nanocomposite surface was positively charged, and electrostatic repulsion occurred between the positive charges in the monoprotonated forms of MY and the positively charged nanocomposite, which reduced the degradation efficiency within 10 min. Similarly, in the basic medium (pH 10.2), the nanocomposite surface was negatively charged. The negative charges because of the OH^–^ ions increased in basic media and so electrostatic repulsion happened between the nanocomposite surface and the dye molecules. As a result of the experiments, 100% degradation efficiency was obtained in 10 min in a neutral medium, and the optimum pH value was determined as 7.1.

### Effect of the Nanocomposite Dosage on Degradation of MY

The catalytic reduction of MY takes place on the active surfaces of the nanocomposite. Therefore, nanocomposite dosage plays an important role in the degradation of MY. The effect of MMT@Fe_3_O_4_@Ag dosage on the degradation of MY was investigated at various dosages ranging from 0.44 to 3.11g/L while keeping pH at 7.1, initial dye concentration at 13.5 mg/L, and NaBH_4_ amount at 0.375 mL. Figure 7b shows that degradation of MY was increased with the dosage of the nanocomposite, which is attributed to the exposed active surface area increasing with the amount of MMT@Fe_3_O_4_@Ag. A higher active surface area accelerated the hydrogen transfer to MY molecules, leading to greater degradation efficiency, and thus, MY was degraded in a shorter time. As a result of the experiments carried out for our study, the optimum nanocomposite dosage was chosen as 2.22 g/L.

### Effect of the Initial Dye Concentration on Degradation of MY

Initial dye concentration studies were performed at concentrations ranging between 9 and 36 mg/L with a dosage of 2.22 g/L MMT@Fe_3_O_4_@Ag, pH 7.1, and 0.375 mL of NaBH_4_. It was noted that with increasing dye concentration, there was an increase in the reaction time required for MR degradation (Figure 7c). This means that the empty active sites on the nanocomposite surface were occupied by MY molecules, but with increasing concentration of MY, the active areas could not degrade enough MY molecules despite the passage of time. This showed that 100% degradation efficiency occurred at 5, 10, and 17 min for dye concentrations of 9, 13.5, and 18 mg/L, respectively, while only 39 and 25% of the degradation reaction occurred within 30 min for dye concentrations of 24 and 36 mg/L, respectively. Within the results obtained, the optimum MY concentration was chosen as 13.5 mg/L.

### Effect of the Amount of NaBH_4_ on Degradation of MY

To show the effect of NaBH_4_ on degradation of MY, the efficiency of the MMT@Fe_3_O_4_@Ag nanocomposite was examined in the range of 0.125–0.425 mL amount of NaBH_4_, and results are presented in Figure 7d. The reaction conditions during the experiments were determined as 2.22 g/L nanocomposite, pH 7.1, and 13.5 mg/L MY. As seen clearly from the graph, there was a noticeable increase in degradation efficiency with the increase in the amount of NaBH_4_. Although the degradation reaction proceeded for 30 min, 0.125 and 0.250 mL of NaBH_4_ performed only 15 and 42% of the reaction, respectively. In addition, when 0.375 and 0.425 mL were used by increasing the amount of NaBH_4_, MY was degraded completely within 5.5 and 10 min, respectively. This situation can be explained as follows: more reactive electrons and hydrogens were produced with the increase in the amount of NaBH_4_, and these were transmitted to MY molecules via the nanocomposite, resulting in faster degradation of MY.^75^ Therefore, the optimum amount of NaBH_4_ was chosen as 0.375 mL for MY degradation in this work.

### HPLC Analysis

**Figure 8:**
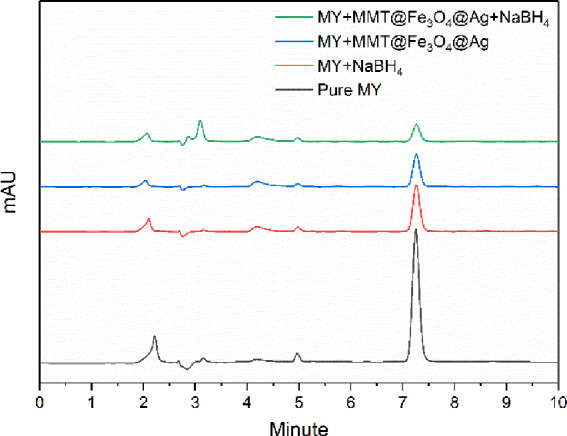
HPLC analysis of the MY solution.

HPLC analysis of MY degradation solutions carried out under optimum conditions was performed, and the chromatograms obtained are presented in Figure 8. The peak observed around 7.25 min in the analysis of 13.5 mg/L MY solution before degradation shows the presence of the MY molecule. The magnitude of this peak decreased slightly only in the presence of NaBH_4_ and only in the presence of MMT@Fe_3_O_4_@Ag. However, as a result of the catalytic degradation process (in the presence of NaBH_4_ + MMT@Fe_3_O_4_@Ag + MY), the decrease in this peak is much higher. In addition, the new peaks observed around 2.85 and 3.09 min in the chromatogram of the catalytic degradation process belong to the new products formed as a result of the degradation reactions. These results prove that MY molecules degrade and transform into new products in the presence of the MMT@Fe_3_O_4_@Ag catalyst and NaBH_4_.^76^

### Stability and Reusability of the MMT@Fe_3_O_4_@Ag Nanocomposite

**Figure 9:**
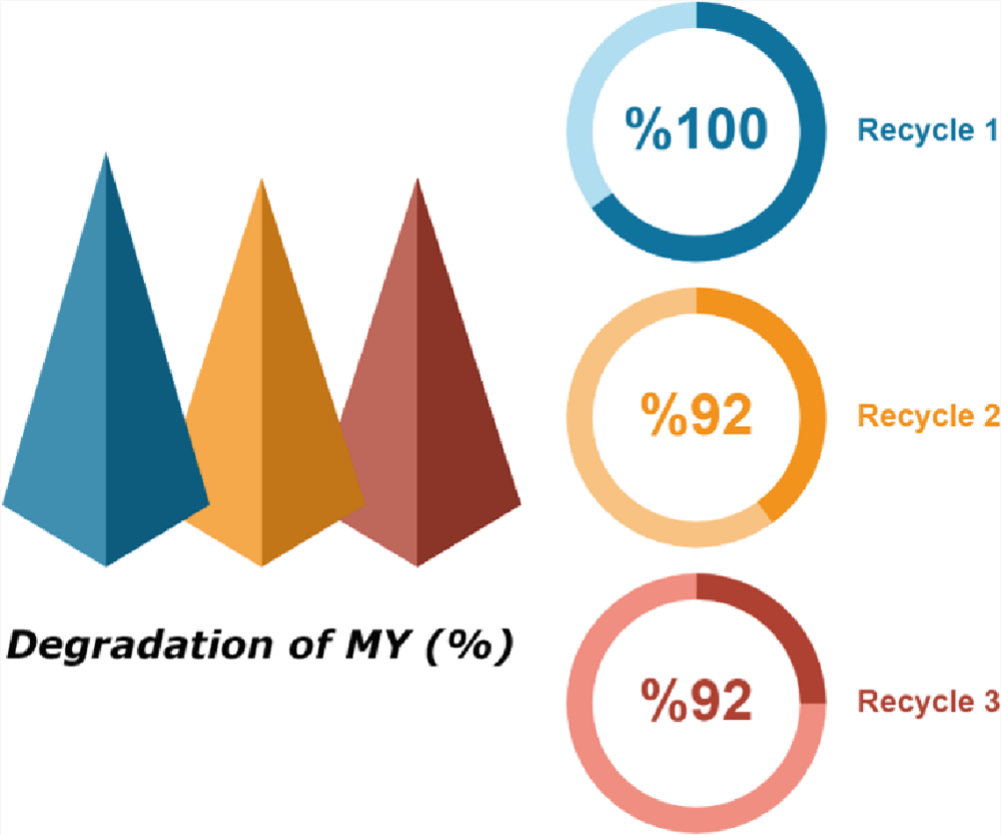
Degradation percentage of MY in three cycles.

The reusability of nanocomposites is an important issue for industrial application. Therefore, reuse of the MMT@Fe_3_O_4_@Ag nanocomposite for degradation of MY was evaluated in this study. When the degradation reaction of MY in the presence of NaBH_4_ was completed, it was observed that the MMT@Fe_3_O_4_@Ag nanocomposite could be easily separated from the reaction medium using an external magnet, and it was reused for the next cycle. As shown in Figure 9, MMT@Fe_3_O_4_@Ag exhibited a catalytic activity of 92% even after recycling for 3 times. These results show that MMT@Fe_3_O_4_@Ag has good catalytic activity without significant loss in its reuse, and this can be explained by the effective immobilization of AgNPs on the Fe_3_O_4_ surface. The stability of the catalyst is important for the degradation of MY. Leaching of Ag and Fe from the nanoparticle reduces the degradation efficiency as it will affect the stability of the particle. The synthesized particles showed good stability and strong degradability for MY removal. The Fe metal leaching amounts of MMT@Fe_3_O_4_ and MMT@Fe_3_O_4_@Ag catalysts were 0.124 and 0.082 mg/L, respectively. These data showed that the MMT@Fe_3_O_4_@Ag catalyst has better reproducibility and stability on MY degradation compared to MMT@Fe_3_O_4_. As a result, the binding of Ag to the MMT@Fe_3_O_4_ surface also reduced the leaching of Fe ions. The Ag leaching for the MMT@Fe_3_O_4_@Ag catalyst was found to be 0.031 mg/L and this value is less than 10% of the loaded Ag amount.^77^

### TON and TOF of the Catalysts

The number of active sites present in the catalysts is equal to the number of moles, and it was estimated by using the catalyst concentration. For this purpose, the surface properties of MMT@Fe_3_O_4_ and MMT@Fe_3_O_4_@Ag and their effectiveness (cycle number (TON) and turnover frequency (TOF)) in the degradation of MY dye were compared. Detailed calculations of the number of moles (13.5 mg/L) of dye, prepared samples, and TON value can be found in the literature.^78^ TON is calculated by the following formula (eq 14):
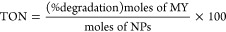
14

The term TOF is expressed as the inverse unit of time (for this study, 10 min), which is actually the number of reaction cycles occurring in a given period of time and is also used to represent the reaction rate, which can be defined as eq 15:
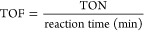
15

TON and TOF values were calculated for MMT@Fe_3_O_4_ and MMT@Fe_3_O_4_@Ag. TON and TOF were found to be 0.337 and 0.033 min^–1^ for MMT@Fe_3_O_4_ and 3.52 and 0.35 min^–1^ for MMT@Fe_3_O_4_/Ag, respectively. The found values indicate that MY on the MMT@Fe_3_O_4_@Ag catalyst significantly increased the degradation efficiency because the TON values for MY degradation were increased from approximately 0.337 to 3.52 on MMT@Fe_3_O_4_@Ag compared to MMT@Fe_3_O_4_.^79^

### Evaluation of ANN Simulation on Experimental Parameters

A suitable model for ANN was run by examining various data sets, including 5 nodes in the input layer, 10 nodes in the hidden layer, and 1 node in the output layer. Input parameters involve solution pH (3–10), MMT@Fe_3_O_4_@Ag nanocomposite dosage (0.44–3.11 g/L), initial dye concentration (9–36 mg/L), the amount of NaBH_4_ (0.125–0.425 mL), and reaction time, which were dispersed among training, validation, and testing in a random manner. The output data set was the selected percentage of MY dye degradation. In this study, the reason MATLAB was preferred in ANN applications was the breadth and flexibility of the research scope.

**Figure 10:**
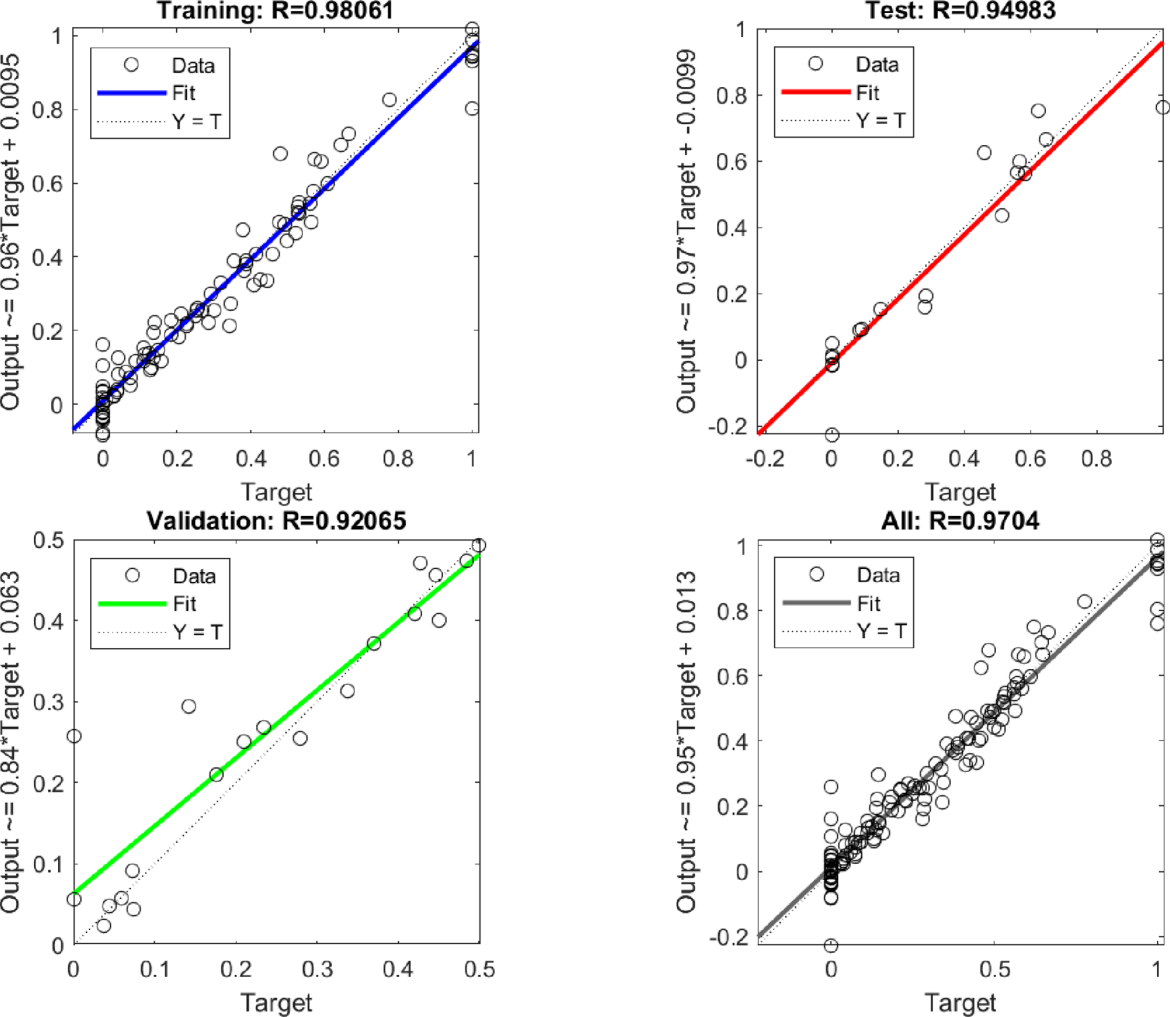
Tests for Linear Regression.

**Figure 11:**
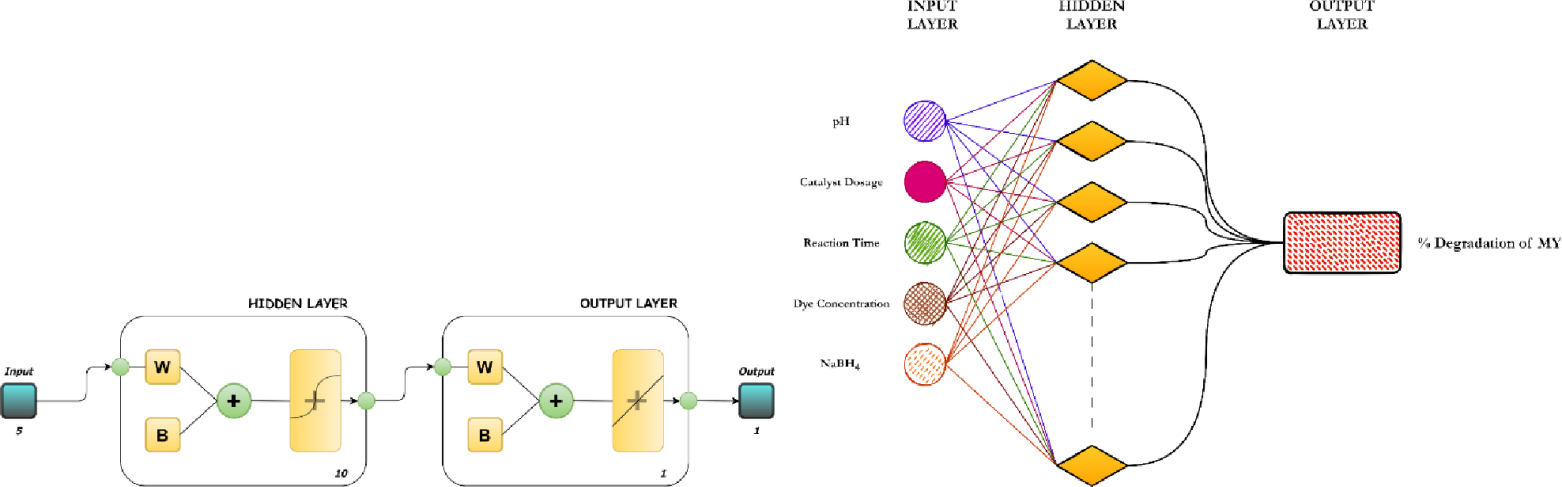
Schematic Diagram of the Optimized ANN Structure.

*R* values for training, validation, and testing were obtained as 0.98, 0.92, and 0.94, respectively (Figure 10). The *R*^2^ and MSE values computed for the training, testing, and validation data sets are presented in Table 3. Figure 11 shows a schematic illustration of the optimized ANN structure.

**Table 3:** Performance Parameters of the Percentage of MY Dye Degradation

parameter	subset	training	test	validation
percentage of MY dye degradation	*R*^2^	0.9604	0.8836	0.8464
	MSE	0.0032	0.0094	0.0052

## Conclusions

In summary, MMT@Fe_3_O_4_ was synthesized via the coprecipitation method with the precipitation of Fe^2+^ and Fe^3+^ ions at pH 10. Then, AgNPs were doped with MMT@Fe_3_O_4_ via the chemical reduction method by adding NaBH_4_ to obtain the MMT@Fe_3_O_4_@Ag nanocomposite. To investigate the catalytic activity of Ag, MMT@Fe_3_O_4_ and MMT@Fe_3_O_4_@Ag were evaluated separately for degradation of MY. Also, the results showed that the MMT@Fe_3_O_4_@Ag nanocomposite exhibited high degradation efficiency for MY compared to MMT@Fe_3_O_4_. Thereafter, parameters including solution pH, catalyst dosage, initial dye concentration, and the amount of NaBH_4_ were examined in the presence of the MMT@Fe_3_O_4_@Ag nanocomposite. Also, optimum reaction conditions were determined as pH 7.1, a catalyst dosage of 2.22 g/L, 13.5 mg/L MY, and an amount of NaBH_4_ of 0.375 mL. In addition, the MMT@Fe_3_O_4_@Ag nanocomposite was easily separated from the reaction medium by using an external magnet after the degradation reaction of MY was completed. Thus, it has been proven that the MMT@Fe_3_O_4_@Ag nanocomposite performed quite well in degradation of MY, which has proven to be carcinogenic. HPLC analysis of MY solutions before and after the degradation processes showed that MY dye was degraded and converted to other products in the presence of NaBH_4_ and the MMT@Fe_3_O_4_@Ag catalyst. Consequently, we showed that the MMT@Fe_3_O_4_@Ag nanocomposite has good potential for environmental remediation through this study. When the ANN results were compared with the experimental results, the high *R* value obtained showed a good prediction of the catalytic degradation process.

## Supporting Information Available

Additional characterization graphs and structural properties of MY (PDF)
