# Supplementary material for: Preparation and Characterization of the MMT@Fe3O4@Ag Nanocomposite for Catalytic Degradation of Methyl Yellow: Reaction Parameters and Mechanism Based on the Artificial Neuron Network
Source: ACS Omega. 2024 Dec 25;10(1):134–46. doi: 10.1021/acsomega.4c02497 (PMC11740151; doi:10.1021/acsomega.4c02497)
Supplement: Supplementary file 1 — ao4c02497_si_001.pdf [file ao4c02497_si_001.pdf]

Supplementary Materials

Preparation and characterization of MMT@Fe_3O_4@Ag nanocomposite for catalytic degradation of methyl yellow: Reaction parameters and mechanism based on artificial neuron network

Turkan Altun[a*], Musa Kazim Acar[a], Ilkay Hilal Gubbuk[b]

[a]Department of Chemical Engineering, Konya Technical University, Konya, 42150, Turkey

[b]Department of Chemistry, Selcuk University, Campus, 42075, Konya, Turkey

* All correspondence should be addressed. E-mail: taltun@ktun.edu.tr

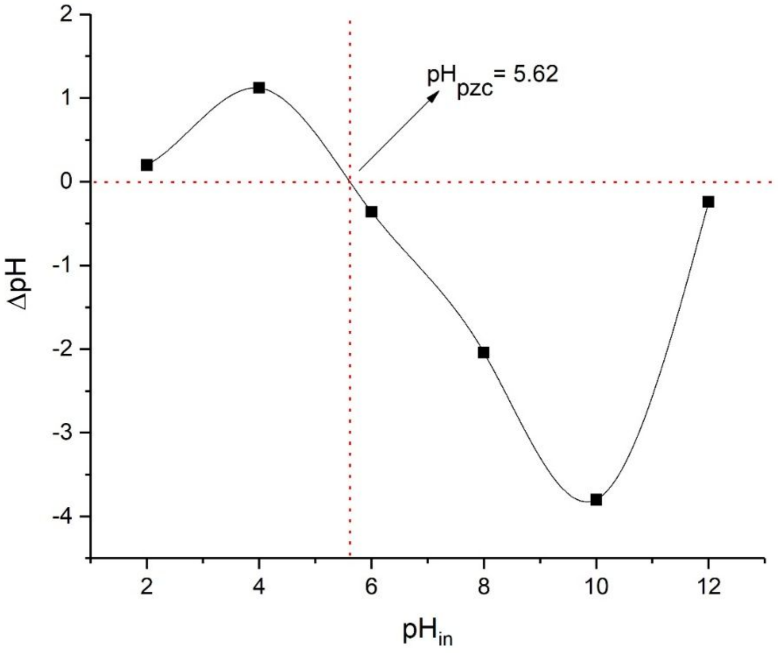

Figure S1. ΔpH versus pH_{in} plot for MMT@Fe_3O_4@Ag nanocomposite

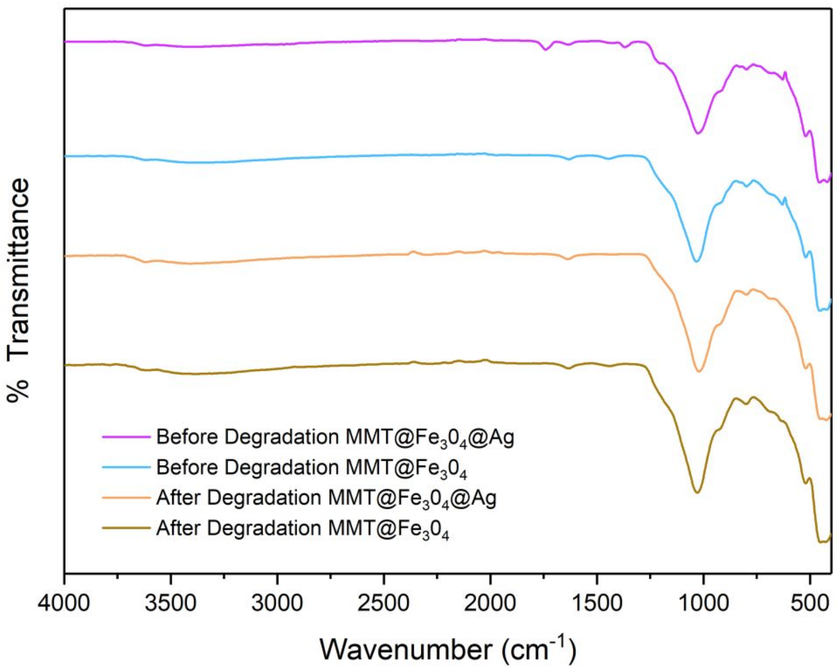

Figure S2. The FTIR spectrum before and after the degradation processes of MMT@Fe_3O_4 and MMT@Fe_3O_4@Ag nanocomposites

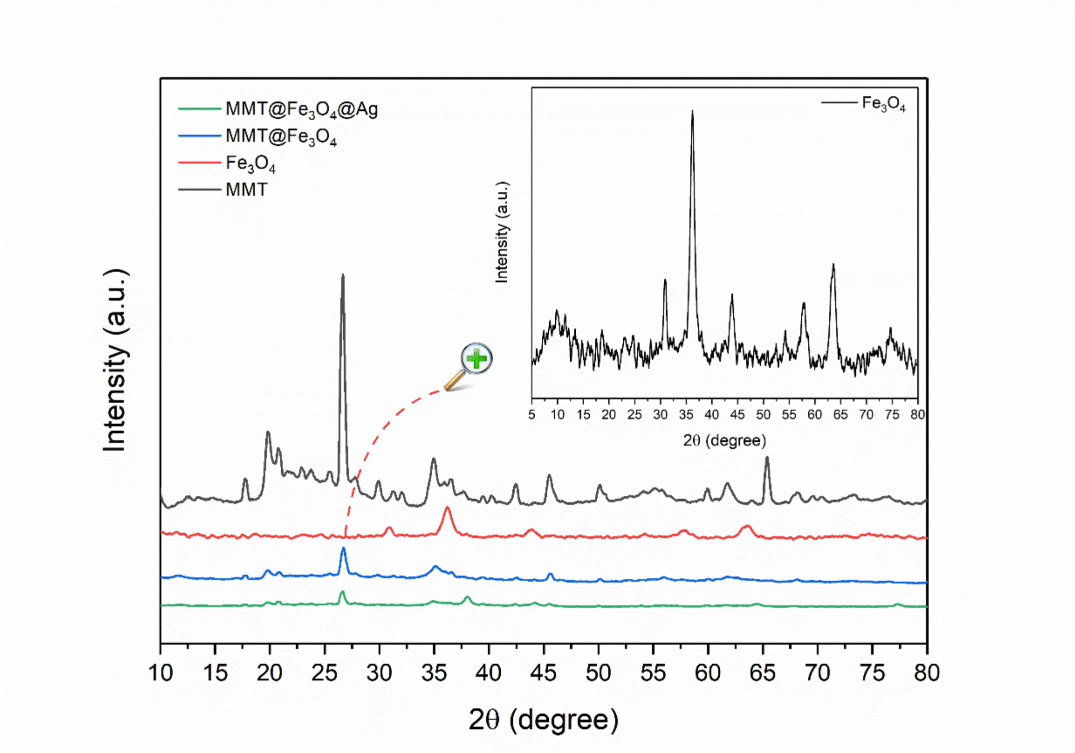

Figure S3. The XRD diffraction of MMT, Fe_3O_4, MMT@Fe_3O_4 and MMT@Fe_3O_4@Ag nanocomposites

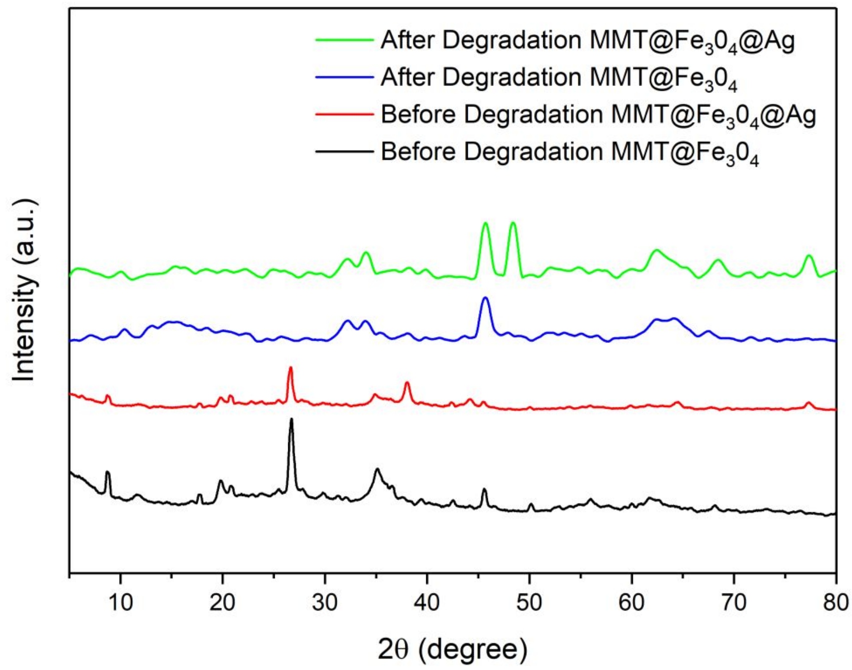

Figure S4. The XRD diffraction before and after the degradation processes of MMT@Fe_3O_4 and MMT@Fe_3O_4@Ag nanocomposites

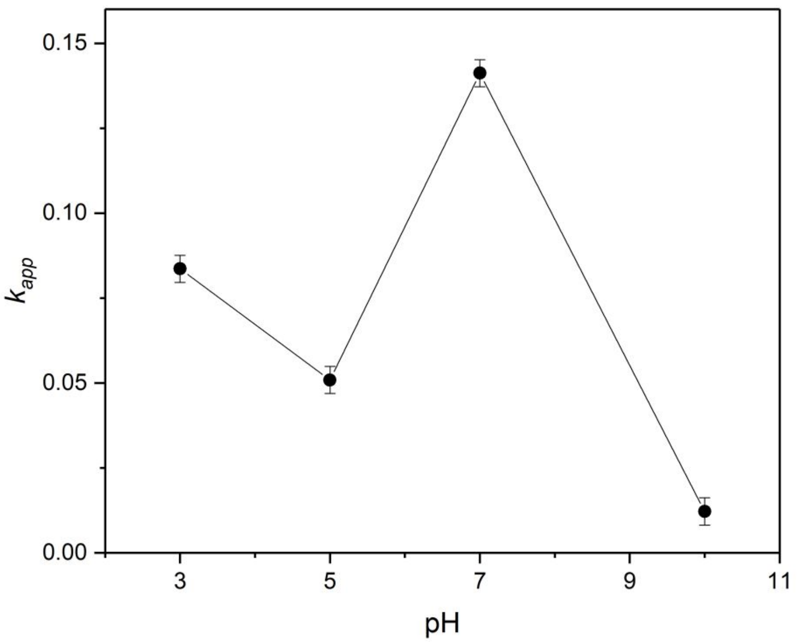

Figure S5. The values of k_{app} depending on pH for degradation of MY

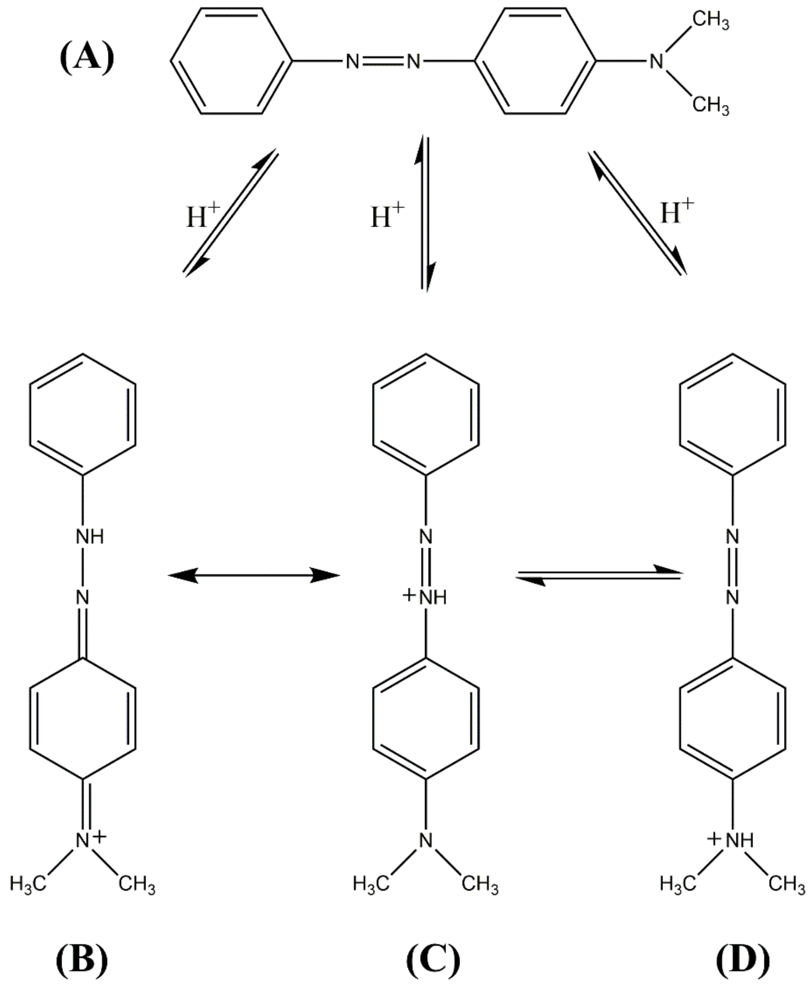

Figure S6. Structure of neutral or base form (A), azonium tautomer (B and C) and ammonium tautomer (D) of MY in aqueous solution

Table S1. Various properties of MY

	Chemical Structure	Class	Type	λ_{max} (nm)	Molecular Weight (g/mol)	Molecular Formula	CAS No.	Physical Appearance
Methyl Yellow	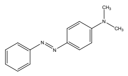	Dispersed	Azo	446	225.295	$C_{14}H_{15}N_3$	60-11-7	Yellow leaf-shaped crystals
Chemical and Physicochemical Properties	Boiling point: 306-371 ℃<br>Melting point: 110-114 ℃<br>Soluble in, chloroform, benzene, alcohol; mineral acids, ether, pyridine, oils and petroleum ether							
